# Phosphoglycerate dehydrogenase inhibition induces p-mTOR-independent autophagy and promotes multilineage differentiation in embryonal carcinoma stem-like cells

**DOI:** 10.1038/s41419-018-0997-8

**Published:** 2018-09-24

**Authors:** Tanveer Sharif, Emma Martell, Cathleen Dai, Mohammad Saleh Ghassemi-Rad, Kristen Lee, Sheila K. Singh, Ian C. G. Weaver, Shashi Gujar

**Affiliations:** 1 Department of Pathology, Dalhousie University, Halifax, NS Canada; 2 Department of Microbiology and Immunology, Dalhousie University, Halifax, NS Canada; 3 Department of Psychology and Neuroscience, Dalhousie University, Halifax, NS Canada; 4 McMaster Stem Cell and Cancer Research Institute, McMaster University, Hamilton, ON Canada; 5 Department of Biochemistry and Biomedical Sciences, Faculty of Health Sciences, McMaster University, Hamilton, ON Canada; 6 Department of Surgery, Faculty of Health Sciences, McMaster University, Hamilton, ON Canada; 7 Department of Psychiatry, Dalhousie University, Halifax, NS Canada; 8 Brain Repair Centre, Dalhousie University, Halifax, NS Canada; 9 Department of Biology, Dalhousie University, Halifax, NS Canada; 10 Centre for Innovative and Collaborative Health Systems Research, IWK Health Centre, Halifax, NS B3K 6R8 Canada

## Abstract

Cancer cells with a less differentiated stem-like phenotype are more resistant to therapeutic manipulations than their differentiated counterparts, and are considered as one of the main causes of cancer persistence and relapse. As such, induction of differentiation in cancer stem-like cells (CSLCs) has emerged as an alternative strategy to enhance the efficacy of anticancer therapies. CSLCs are metabolically distinct from differentiated cells, and any aberration from the intrinsic metabolic state can induce differentiation of CSLCs. Therefore, metabolism-related molecular targets, with a capacity to promote differentiation within CSLCs, are of therapeutic importance. Here, we demonstrate that phosphoglycerate dehydrogenase (PHGDH), an essential enzyme catalyzing the synthesis of amino acid serine, is important for maintaining the poorly differentiated, stem-like state of CSLCs. Our data shows that PHGDH deficiency impairs the tumorsphere formation capacity in embryonal carcinoma stem-like cells (ECSLCs), breast cancer stem-like cells (BCSLCs) and patient-derived brain tumor-initiating cells (BTICs), which is accompanied by the reduced expression of characteristic stemness-promoting factors, such as Oct4, Nanog, Sox-2, and Bmi-1. Mechanistically, PHGDH deficiency in ECSLCs promotes differentiation to various lineages via degradation of Oct4 and by increasing the stability of differentiation marker β3-tubulin. Furthermore, PHGDH inhibition promotes p-mTOR independent but Beclin-1-dependent autophagy, independent of apoptosis. When studied in combination, the inhibition of both PHGDH and p-mTOR in ECSLCs causes further augmentation of autophagy, and additionally promotes apoptosis, demonstrating the clinical applicability of PHGDH-based manipulations in cancer therapies. Recapitulating these in vitro findings in CSLC models, the intratumoral PHGDH expression in patient-derived tumors is positively correlated with the mRNA levels of stemness factors, especially Oct4, and cancer patients co-expressing high levels of PHGDH and Oct4 display significantly lower survival than those with low PHGDH/Oct4 co-expression. Altogether, this study identifies a clinically-relevant role for PHGDH in the regulation of stemness-differentiation axis within CSLCs.

## Introduction

A well-established feature of cancer cells is their enhanced capacity to proliferate^1^. In order to maintain this aberrant growth rate, cancer cells have increased energy requirements and are known to reprogram metabolic pathways to sustain higher demand for cellular building blocks, such as proteins and nucleotides^2^. Serine is a non-essential amino acid (NEAA) that is used in the synthesis of proteins and nucleic acids and is rapidly consumed by cancer cells^3,4^. Because of this, the serine biosynthesis pathway is often upregulated in cancer cells. Phosphoglycerate dehydrogenase (PHGDH), the enzyme which catalyzes the first step of the serine biosynthesis pathway, has been shown to be genomically amplified in many breast cancers and melanomas^5,6^. High levels of PHGDH have been associated with enhanced proliferation and poor prognosis in various types of cancers, and cancer cells that harbor high levels of PHGDH have been shown to be more susceptible to PHGDH inhibition^5,7^.

Despite the recent discoveries enhancing the available options for cancer treatment, relapse from this disease remains a major hurdle in clinics. It is now being recognized that tumors are comprised of heterogeneous populations of cells which contain cells with both differentiated as well as stem-like features^8,9^. This intratumoral heterogeneity is an important determinant of cancer relapse, as the constituting cancer stem-like cells (CSLCs) are linked with greater resistance to various cancer treatments^8,10–12^. These CSLCs are characterized by different growth characteristics, degree of differentiation, and expression of cell surface markers^9,10,13^. Interestingly, markers used to identify CSLC populations can vary for different types of cancers. For example, high expression of the cell surface marker CD44 and low expression of CD24 are commonly used as markers of breast cancer stem-like cells (BCSLCs)^14,15^, whereas high expression of CD133 is a standard marker for identifying CSLCs in many brain malignancies^16^. Moreover, CSLCs from various origins may display aberrant expression of genes usually expressed in embryonic stem cells (such as Oct4, Nanog, Sox-2, Myc, KLF-4, and Lin28b), and expression of these embryonal stem cell (ESC) signature genes in tumors is associated with a poorly differentiated state and enhanced aggressiveness^17,18^. In addition to their poorly differentiated state and expression of surface markers, CSLCs are also characterized by their unlimited replicative potential, and their ability to give rise to both daughter CSLC progeny as well as differentiated cancer cells which comprise the bulk of the tumor^8^. Interestingly, the self-renewal and tumorigenicity of CSLCs can be suppressed by promoting their differentiation^15,19,20^, and thus the strategies promoting differentiation within CSLCs bear therapeutic promise. Recently, autophagy, a catabolic degradation process influenced by cellular energy and metabolic perturbations, was identified as a crucial regulator of self-renewal and differentiation within stem cells^21–23^. Thus, it may be possible to target CSLCs by developing autophagy-based differentiation-inducing therapies. This approach requires an understanding of the genes and pathways that link stemness within CSLCs and autophagy in the context of cell metabolism. In this study, we identified that serine-metabolizing enzyme PHGDH plays an important role in the maintenance of self-renewal and poorly differentiated state of the CSLCs through modulation of autophagy.

Here, we report that the inhibition of PHGDH expression in various CSLCs severely inhibits their tumorsphere formation capacity, and demonstrate the requirement of PHGDH in maintenance of the characteristic stemness feature of CSLCs. Out of different CSLCs tested, the embryonal carcinoma stem-like cells (ECSLCs) NT2/D1^24,25^ harbor significantly higher levels of PHGDH as compared to the differentiated, PHGDH-overexpressing (PHGDH^High^) MDA-MB-468 breast cancer cells, as well as CSLCs of human mammary epithelial origin (HMLER^shECad^)^15^, and three different patient-derived brain tumor-initiating cells (BTICs)^26^. Moreover, NT2/D1 ECSLCs were the only cells identified in our screen to co-express higher levels of both PHGDH and the master pluripotency factor Oct4. This positive correlation between PHGDH and Oct4 expression in vitro is reflected in human tumors and corresponds to patient prognosis, as the survival probability for patients with tumors concomitantly expressing high levels of PHGDH and Oct4 is significantly lower than those with concurrently low expression of PHGDH and Oct4. Consequently, ablation of PHGDH expression in NT2/D1, HMLER^shECad^ and BTICs severely inhibited their tumorsphere formation capacity and promoted differentiation of ECSLCs to multiple lineages. Inhibition of Oct4 in PHGDH^High^ ECSLCs also inhibited PHGDH mRNA expression, demonstrating that once ECSLCs lose their pluripotent capacity they no longer require high levels of PHGDH. Further mechanistic analysis revealed that the inhibition of ECSLC growth and self-renewal following PHGDH inhibition or depletion occurs independent of apoptosis. Instead of programmed cell-death, PHGDH KD promotes senescence and induces autophagy in a p-mTOR-independent and Beclin-1-dependent manner.

## RESULTS

### Human ECSLCs NT2/D1 co-express high levels of PHGDH and Oct4

**Fig. 1:**
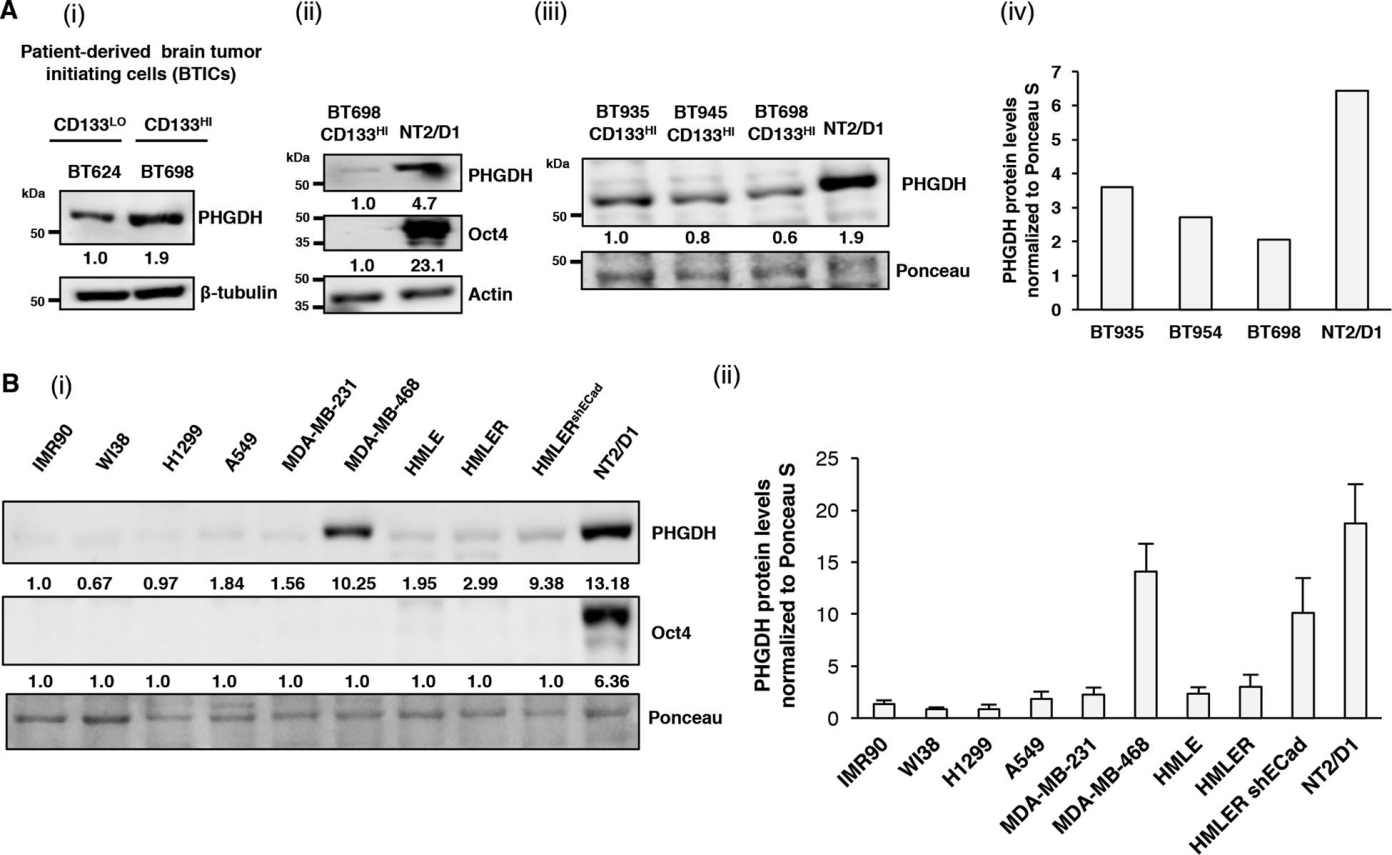
NT2/D1 ECSLCs co-express high levels of PHGDH and Oct4. **a** (i) CD133^Low^ BT624 and CD133^High^ BT698 BTICs were subjected to western blot (WB) analysis for PHGDH.(ii) CD133^High^ BT698 BTICs and NT2/D1 cells were subjected to WB analysis for PHGDH and Oct4. (iii) Levels of PHGDH were compared between BTICs derived from three individual patients (BT935, BT954, and BT698) and NT2/D1 ECSLCs by WB analysis. (iv) Quantification of PHGDH protein levels normalized to Ponceau stain. **b** (i) IMR90, WI38, H1299, A549, MDA-MB-231, MDA-MB-468, HMLE, HMLE-Ras (HMLER), HMLER shE-Cadherin (HMLER^shECad^), and NT2/D1 cells were subjected to WB analysis for PHGDH and Oct4.(ii) Quantification of PHGDH protein levels normalized to Ponceau S.

To understand the relationship between PHGDH and the stemness of CSLCs, we compared the levels of PHGDH in primary, patient-derived CD133+ glioblastoma (GBM) cells^16^. It has been previously shown that CD133^High^ GBM cells represent more stem-like brain tumor initiating cells (BTICs) than CD133^Low^ GBM cells^26^. In line with these data, through a direct comparison, we found that CD133^High^ BTICs BT698 expressed relatively higher levels of PHGDH than CD133^Low^ GBM cells BT624 [Fig.1a(i)]. Although patient-derived CD133^High^ BTICs display bona fide CSLC properties^14^, we found that they do not express detectable levels of master stemness-regulating factor Oct4 [Fig. 1a(ii)]. Therefore, to study the relationship between PHGDH and Oct4, we used a human teratocarcinoma cell line NT2/D1, which expresses high levels of pluripotency factor Oct4^22^. We found that NT2/D1, expressing high levels of Oct4 (Oct4^High^), also expressed higher levels of PHGDH, as compared to three different patient-derived CD133^High^ BTICs [Fig. 1a(ii), (iii) & (iv)].

We next compared the expression of PHGDH across multiple non-transformed and cancer cell lines from different lineages, including cell lines which are known to harbor high levels of PHGDH^5,28^ (non-transformed: IMR90 and WI38; lung carcinoma: H1299 and A549; breast carcinoma: MDA-MB-231 and MDA-MB-468) and a human breast cancer progression model that was developed to directly compare the differential aspects of “normal” (HMLE), “cancerous” (HMLER) or CSLC (HMLER^shECad^) phenotypes^15^ with the NT2/D1 ECSLC line. Interestingly, we found that the levels of PHGDH were drastically higher in HMLER^shECad^ BCSLCs compared to their non-CLSC malignant (HMLER) as well as normal, non-transformed (HMLE) counterparts (Fig. 1b). However, we found that NT2/D1 cells were the only cell line to co-express high levels of Oct4 and PHGDH [Fig. 1b (i) and (ii)] within the tested set of cells, and thus represented a suitable model to investigate the interplay between PHGDH and Oct4. Therefore, PHGDH^High^, Oct4^High^ NT2/D1 ECSLCs were used as a tool to understand the interplay between PHGDH and Oct4.

Altogether, our findings demonstrating that CSLCs from various origins (HMLER^shECad^, CD133^high^ BTICs, and NT2/D1) express more PHGDH than their cancerous non-stem-like cells counterparts (HMLER, CD133^Low^ GBM) highlight the role of PHGDH in stemness. These results compliment the previous report by Samanta et al.^28^ where they showed that PHGDH expression is induced during the Hypoxia related enrichment of BCSCs.

### High intra-tumoral co-expression of PHGDH and Oct4 predicts poor survival in cancer patients

**Fig. 2:**
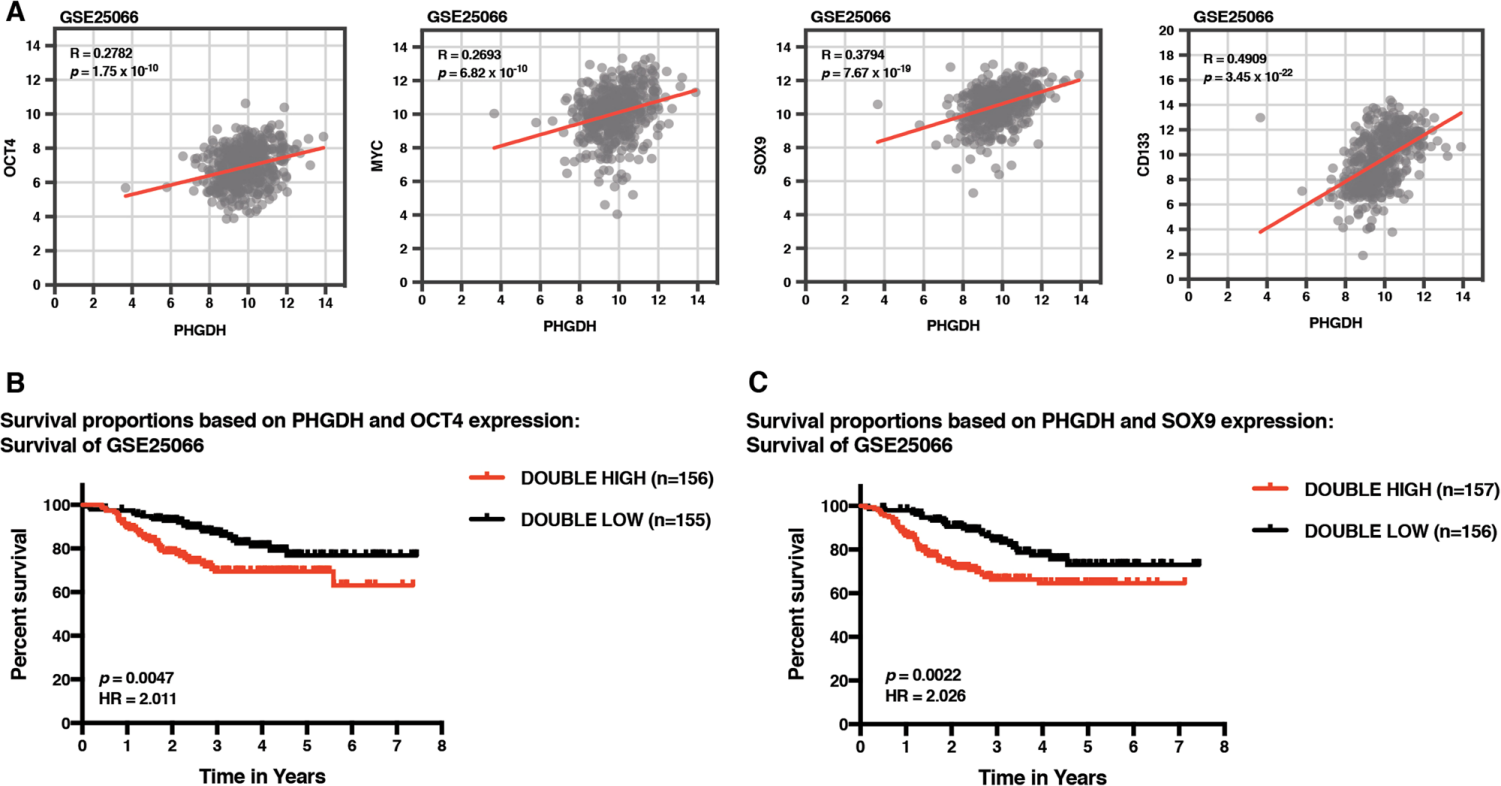
Relationship between PHGDH and stemness-regulating factors is reflected in human tumors, and is indicative for patient prognosis. **a** GSE25066 database from breast tumors was analyzed for the correlation between PHGDH and OCT4, MYC, SOX9, and CD133. **b** Kaplan Meier survival analysis of public patient biopsy datasets based on high expression of both PHGDH and OCT4 (Double High) versus low expression of both PHGDH and OCT4 (Double Low) (cutoff = median expression) from breast tumors (GSE25066). **c** Kaplan Meier survival analysis of public patient biopsy datasets based on high expression of both PHGDH and SOX9 (Double High) versus low expression of both PHGDH and SOX9 (Double Low) (cutoff = median expression) from breast tumors (GSE25066)

To understand the translational significance of our in vitro findings in human tumor biology, we analyzed a publicly available gene expression array dataset (GSE25066) from breast cancer patients, to determine whether PHGDH levels correlate with the expression of stemness factors in patient-derived tumors. We observed a positive and statistically significant correlation between the levels of PHGDH with the expression of the major pluripotency transcription factor Oct4 (*R* = 0.2782) as well as other markers of cancer stemness, including MYC (*R* = 0.2693), SOX9 (*R* = 0.3794), and CD133 (*R* = 0.4909)^12,29–31^ (Fig. 2a). These patient-derived tumor datasets further supported our in vitro findings regarding the association between the serine-metabolizing enzyme PHGDH and the cancer stemness-maintaining factors.

Next, we wished to understand the clinical relevance of the correlation between PHGDH and stemness in human cancers. Therefore, we analyzed the relationship between the co-expression of PHGDH and Oct4 or SOX9, and the clinical outcome from cancer. We observed that the survival within the patients with tumors co-expressing high levels of PHGDH and Oct4 was significantly lower than that of the patients with the low co-expression (GSE25066: *p* = 0.0047; HR = 2.011) and similarly, we found that tumors from patients co-expressing high levels of PHGDH and SOX9 had significantly poorer prognosis than those expressing low levels of both PHGDH and SOX9 (*p* = 0.0022; HR = 2.026) (Fig. 2b, c). Taken together, these data highlight a therapeutic implication for PHGDH and stemness in cancer, and further advocate for the need to understand the mechanistic aspects of this clinically relevant association.

### PHGDH inhibition suppresses characteristic stemness features within CSLCs

**Fig. 3:**
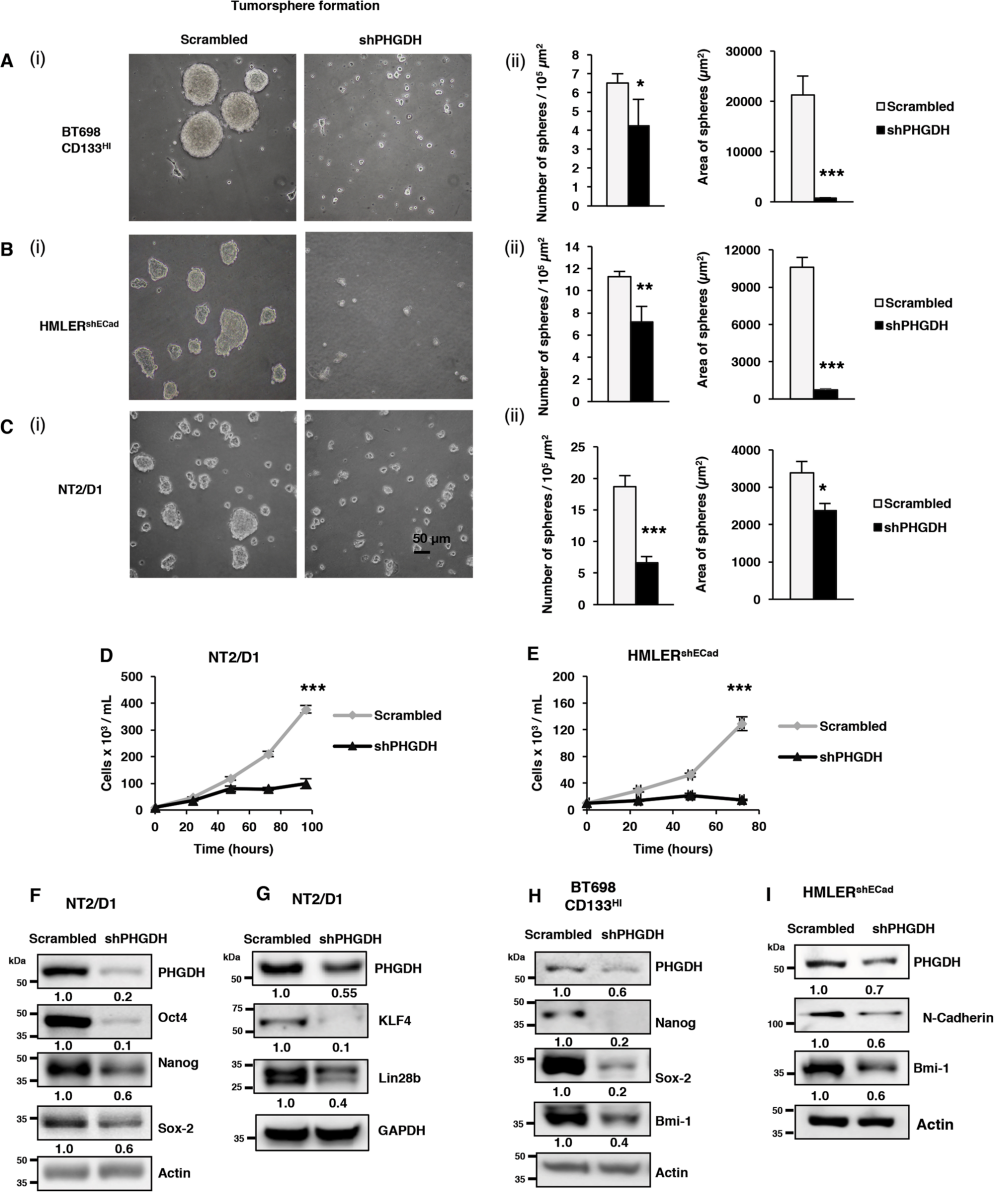
PHGDH is required to maintain stemness and self-renewal in CSLCs. **a** (i) CD133^High^ BT698 BTICs, **b** (i) HMLER^shECad^, and **c** (i) NT2/D1 cells with either scrambled control or PHGDH shRNA were subjected to 3D tumorsphere formation assay, and (ii) average number of tumorspheres (≥50 µm in diameter) per 10^5^ µm^2^ plate surface area and average area of spheres (µm^2^) was analyzed. **d** NT2/D1 and **e** HMLER^shECad^ with either scrambled control or PHGDH shRNA were stained with trypan blue and counted to determine the number of viable cells after 24, 48, and 72 h. **f** NT2/D1 cells with either scrambled control or PHGDH shRNA were subjected to western blot (WB) analysis for Oct4, Nanog and Sox-2, **g** KLF4 and Lin28b. **h** CD133^High^ BT698 BTICs with either Scrambled control or PHGDH shRNA were subjected to WB analysis for Nanog, Sox-2 and Bmi-1. **i** HMLER^shECad^ cells with either scrambled control or PHGDH shRNA were subjected to WB analysis for N-Cadherin and Bmi-1. *p ≤ 0.05; **p ≤ 0.01; ***p ≤ 0.001

Previously, Samanta et al.^28^ have demonstrated that PHGDH is required for the maintenance of hypoxia-induced breast cancer stem cells (BCSCs). However, the role of PHGDH in regulating stemness factors, self-renewal and maintaining the poorly differentiated state remains unexplored. To probe this, we investigated the effect of PHGDH depletion in several CSLC models including; primary, patient-derived BTICs, generated from surgically resected glioblastomas and characterized with the expression of CD133^16^, widely used breast CSLCs (BCSLCs)^14^, and human ECSLCs^24,25^. To determine whether PHGDH inhibition has any effect on the stemness and self-renewal of CSLCs, we measured the tumorsphere formation capacity of BT698 CD133^High^ BTICs, HMLER^shECad^ BCSLCs, and NT2/D1 ECSLCs following the KD of PHGDH^12,20^. We found that PHGDH KD inhibited the self-renewal capacity of BT698 CD133^High^ (Fig. 3a), HMLER^shECad^ (Fig. 3b) and NT2/D1 (Fig. 3c) as evidenced by a statistically significant decrease in the number of tumorspheres formed (≥50 µm in diameter), which were also significantly smaller in size, as compared to those with the respective scrambled control shRNA^12,20^. In addition to investigating the effect of PHGDH depletion on stemness features, we also analyzed the growth rate following PHGDH KD in NT2/D1 cells through two distinct shRNA clones and treatment with PHGDH inhibitor (Fig. 3d; Fig. S1A). We found that the inhibition of PHGDH with either shRNA or pharmacological agent, CBR-5884, significantly decreased the growth-rate of NT2/D1 cells in a dose-dependent manner (Fig. 3d; Fig. S1A & S1B) as measured by trypan blue exclusion method. Similar results were observed following PHGDH KD, using two distinct clones, in HMLER^shECad^ BCSLCs (Fig. 3e; Fig. S1C). Furthermore, shPHGDH KD decreased the expression of widely acknowledged stemness-maintaining factors such as Oct4, Nanog, Sox-2, KLF4, Lin28B, Bmi-1, and N-cadherin in the respective CSLC models (Fig. 3f–h)^14,17,18,29,31–33^. Similar effects on stemness-maintaining factors were also observed using an additional PHGDH shRNA clone in NT2/D1 and HMLER^shECad^ (Fig. S1D & S1E), as well as transient small-interfering RNA (siRNA)-mediated KD (Fig. S1F) and pharmacological inhibition (CBR-5884) (Fig. S1G) of PHGDH in NT2/D1 ECSLCs. These data conclusively demonstrate that the inhibition of PHGDH hampers the characteristic features of stemness within CSLCs. These findings complement a previous report by Samanta et al.^28^ which has shown that PHGDH plays a very important role in hypoxia-induced BCSCs enrichment and PHGDH inhibition decreases hypoxia-induced BCSCs enrichment. Our results now show that PHGDH expression is critical for maintaining the pluripotency and self-renewal of CSLCs from various origins, including patient-derived BTICs. Altogether, these findings unveil a previously unexplored role of PHGDH in the regulation of self-renewal and stemness factors (KLF4, Oct4, Nanog, Sox-2, and Lin28B) in cancer stem-like cells.

### PHGDH inhibition promotes ubiquitination and proteasomal degradation of OCT4 in ECSLCs

**Fig. 4:**
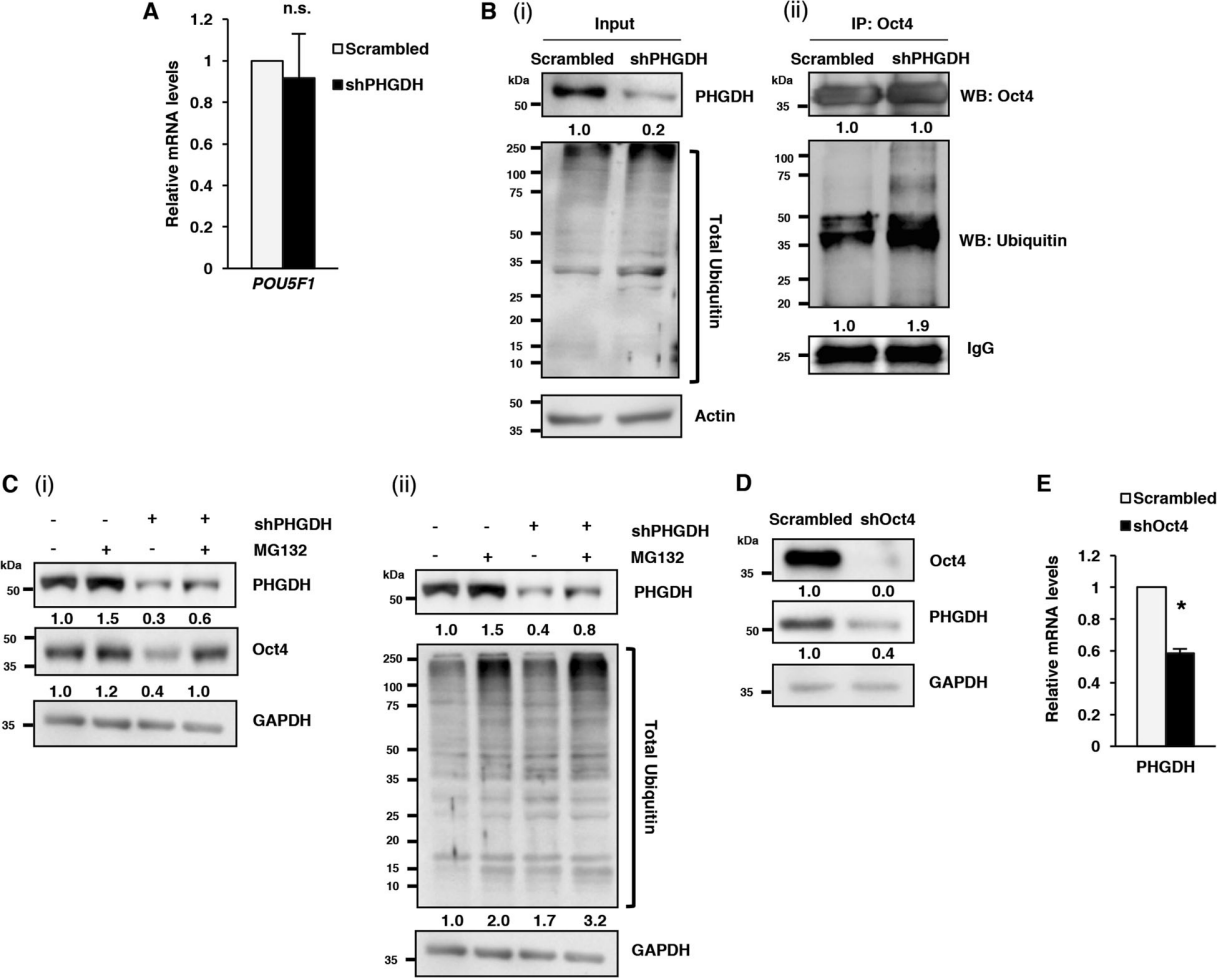
PHGDH inhibition regulates Oct4 at post-translational levels in ECSLCs. **a** NT2/D1 cells, with scrambled control or PHGDH shRNA, were subjected to qRT-PCR analysis for *POU5F1*(Oct4) mRNA levels. **b** NT2/D1 cells, with scrambled control or PHGDH shRNA, were subjected to (i) WB analysis for total levels of ubiquitin, and (ii) immunoprecipitation (IP) using anti-Oct4 antibody followed by WB analysis for specific ubiquitination of Oct4. **c** NT2/D1 cells, with scrambled control or PHGDH shRNA, were treated with MG132, and subjected to WB analysis for (i) Oct4, and (ii) total levels of ubiquitin. **d**, **e** NT2/D1 cells, with scrambled control or Oct4 shRNA, were subjected to **d** WB and **e** qRT-PCR analysis for PHGDH/*PHGDH*, respectively. * *p* ≤ 0.05; **p ≤ 0.01; ***p ≤ 0.001

To further understand how PHGDH maintains stemness in ECSLCs, we sought to dissect how PHGDH inhibition reduced the expression of Oct4 (as shown in Fig. 3). We found that shPHGDH- or siPHGDH-mediated KD had no effect on the mRNA levels of Oct4, even after 72 h post siRNA transduction (Fig. 4a; Fig. S2A). Therefore, we postulated that PHGDH must regulate Oct4 expression at the post-transcriptional level. First, we observed that there was no apparent change in total ubiquitinated protein levels following PHGDH KD (Fig. S2B). Further analysis of specific ubiquitination of Oct4 by targeted immunoprecipitation of Oct4, followed by ubiquitination analysis, showed that the specific ubiquitination of Oct4 increased following PHGDH KD in ECSLCs [(Fig. 4b(ii)]. These results indicate that PHGDH KD promotes the ubiquitination of Oct4, and possibly targets it for proteasomal degradation. In support of this hypothesis, we found that treatment of cells with PHGDH KD with the proteasomal inhibitor MG132 rescued Oct4 levels in ECSLCs [Fig. 4c (i)]. We also found that combination of PHGDH KD and MG132 treatment further enhanced the levels of total ubiquitinated proteins in ECSLCs [Fig. 4c (ii)]. Interestingly, PHGDH KD increased the mRNA expression of proteasomal subunits (PSMA5, PSMB1, PSMB5, and PSMD4) (Fig. S2C), indicating a possible upregulation of ubiquitin-proteasomal system (UPS) in ECSLCs following PHGDH KD. Collectively, these results suggest that PHGDH inhibition upregulates Oct4 degradation through post-translational ubiquitination and proteasomal degradation.

Oct4 is a key factor for maintaining the stemness and poorly differentiated form of ECSLCs, and as such, Oct4 levels are tightly controlled and either upregulation or downregulation of Oct4 can promote their differentiation into various lineages^22,34^. To understand how PHGDH is regulated during the differentiation of ECSLCs, we induced differentiation in NT2/D1 cells by knocking-down Oct4 and measured the expression of PHGDH. We found that depletion of Oct4 reciprocally decreases the expression of PHGDH (Fig. 4d), indicating that once ECSLCs lose their stem-like features, they no longer require high expression of PHGDH. Supporting this conclusion, we also found that Oct4 KD decreased the mRNA levels of PHGDH (Fig. 4e) in ECSLCs, which is not surprising given that Oct4 is a major transcription factor known to regulate the transcription of many genes.

### PHGDH KD increases stability of β3-tubulin and promotes multilineage differentiation in ECSLCs

**Fig. 5:**
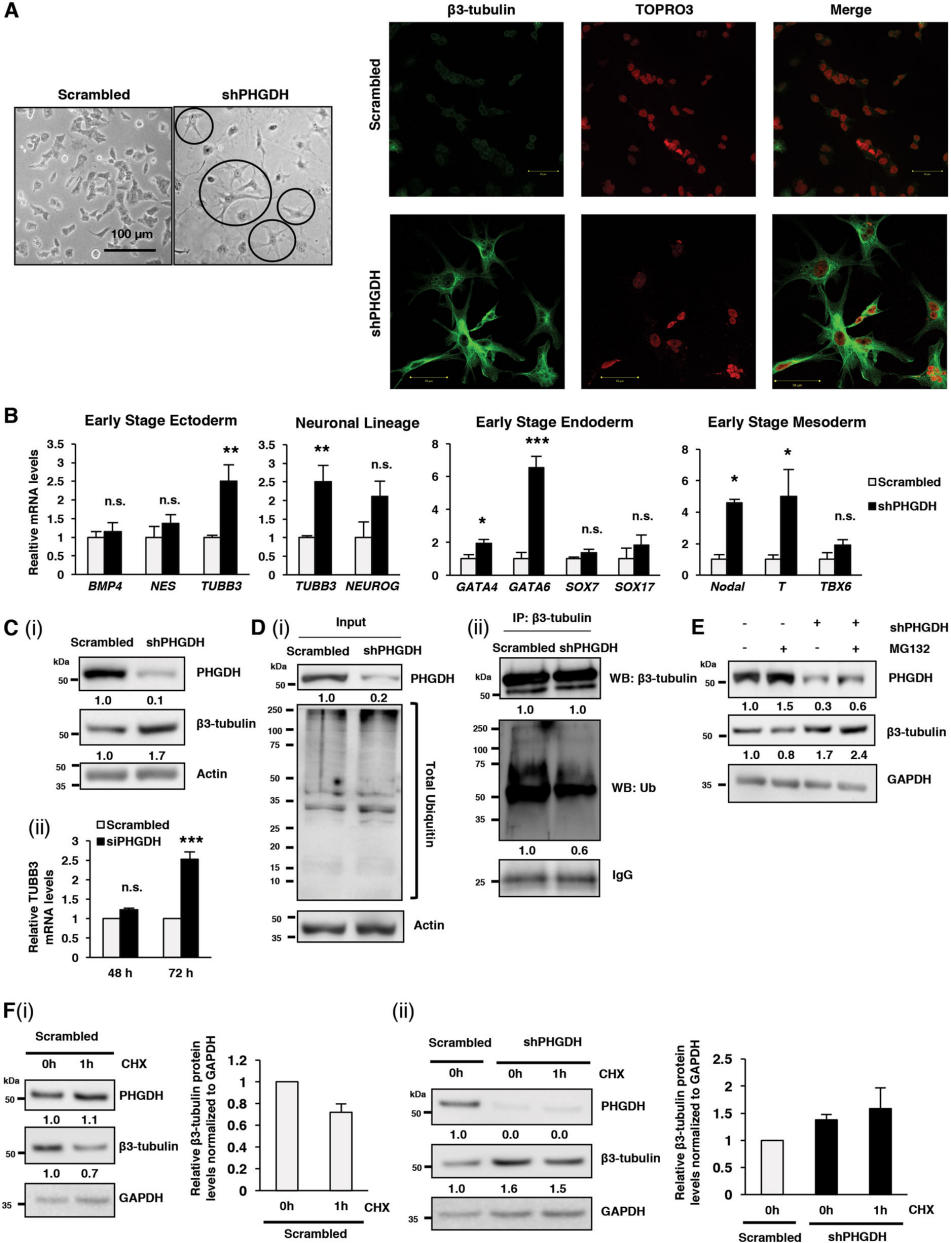
PHGDH KD promotes differentiation of ECSLCs through differential-ubiquitination of β3-tubulin. NT2/D1 cells, with scrambled control or PHGDH shRNA, were subjected to **a** microscopic analysis to compare morphology; and immunofluorescence for β3-tubulin and analyzed by confocal microscopy; or **b** qRT-PCR analysis for differentiation markers from early-stage ectoderm (*BMP4, NES, TUBB3)*, neuronal lineage (*TUBB3, NEUROG1)*, early-stage endoderm (*GATA4, GATA6, SOX7, SOX17)*, and early-stage mesoderm (*Nodal, T, TBX6*). **c** (i) NT2/D1 cells, with scrambled control or PHGDH shRNA, were subjected to western blot (WB) analysis for β3-tubulin protein levels. (ii) NT2/D1 cells, with scrambled control or PHGDH siRNA, were subjected to qRT-PCR analysis for *TUBB3* mRNA levels 48 and 72 h post transfection. **d** NT2/D1 cells, with scrambled control or PHGDH shRNA, were subjected to (i) WB analysis for total levels of ubiquitin, and (ii) immunoprecipitation (IP) using anti-β3-tubulin antibody followed by WB analysis for specific ubiquitination of β3-tubulin. **e** NT2/D1 cells, with scrambled control or PHGDH shRNA, were treated with MG132 and subjected to WB analysis for β3-tubulin. **f** NT2/D1 cells, with (i) scrambled control or (ii) PHGDH shRNA were treated with the translation inhibitor cyclohexamide (CHX) for 1 h, and then subjected to WB analysis for β3-tubulin. *p ≤ 0.05; **p ≤ 0.01; ***p ≤ 0.001

When ECSLCs lose their pluripotent potential due to the loss of Oct4, they may undergo differentiation into various lineages^22,35^; however, the role of PHGDH in regulating differentiation has remained poorly understood. Hence, next we characterized the differentiation status of ECSLCs following PHGDH KD-mediated loss of Oct4. We found that, morphologically, PHGDH KD in NT2/D1 ECSLCs cells induced dendrite-like outgrowths that are characteristic of a differentiated cellular phenotype [Fig. 5a]. These morphological changes were confirmed using immunofluorescent staining of differentiation marker β3-tubulin, where PHGDH KD cells expressed more β3-tubulin compared to scrambled control cells, and this β3-tubulin was localized to the periphery of the cells in the dendrite-like structures [Fig. 5a]. When analyzed for the mRNA levels of differentiation markers from early-stage ectoderm (*BMP4, NES*, β3-tubulin/*TUBB3*), neuronal lineage (β3-tubulin/*TUBB3*, *NEUROG*), early-stage endoderm (*GATA4, GATA6, SOX7, SOX17*), and early-stage mesoderm (*NODAL*, T Brachyury transcription factor/*T, TBX6*)^35^ lineages, we found that PHGDH KD significantly upregulated the transcription of β3-tubulin/*TUBB3*, *GATA4, GATA6, NODAL* and T (Fig. 5b). Corresponding to this qRT-PCR data, stable or transient PHGDH KD in NT2/D1 cells increased β3-tubulin protein levels [Fig. 5c (i)] and β3-tubulin/*TUBB3* transcript levels, respectively [Fig. 5c (ii)]. Together, these results suggested that PHGDH inhibition promotes differentiation in ECSLCs.

To characterize the mechanism by which PHGDH KD regulates differentiation and β3-tubulin expression in ECSLCs, we also analyzed the post-translational modification of β3-tubulin following PHGDH KD in NT2/D1 cells. In contrast to Oct4 (as shown in Fig. 4), PHGDH KD decreased the specific ubiquitination of β3-tubulin [Fig. 5d(ii)], indicating that, in addition to upregulating its transcription, PHGDH KD stabilizes β3-tubulin at post-translational level, which was further enhanced following treatment with proteasomal inhibitor MG132 (Fig. 5e). Of note, NT2/D1 cells show decreased β3-tubulin protein levels following treatment with a translation inhibitor cyclohexamide (CHX; decreased ~30% [Fig. 5f (i)], suggesting that in the absence of on-going translation, β3-tubulin protein levels decline. However, following similar 1 h of CHX treatment, NT2/D1 cells with PHGDH KD displayed relatively stable levels of β3-tubulin (Fig. 5f [ii]). Overall, these findings indicate that PHGDH deficiency-induced differentiation in ECSLCs is accompanied by enhanced mRNA transcription as well as increased stability of β3-tubulin protein through reduced post-translational protein degradation.

In summary, our results indicate that PHGDH deficiency leads to loss of pluripotency that ultimately promotes multilineage differentiation in CSLCs. Samanta et al.^28^ also have demonstrated that PHGDH deficiency suppresses the accumulation of hypoxia-induced BCSCs; however, our findings reveal a novel mechanism wherein the loss of stemness due to PHGDH deficiency is accompanied by mutlilineage differentiation. These findings are of particular interest, as in recent years, the induction of differentiation in CSLCs has been demonstrated to increase sensitivity to chemotherapy and thus has emerged as a novel therapeutic strategy. Altogether, our findings unveil a novel link between PHGDH and differentiation that may have important clinical implications.

### PHGDH inhibition promotes autophagy (not apoptosis) in ECSLCs

**Fig. 6:**
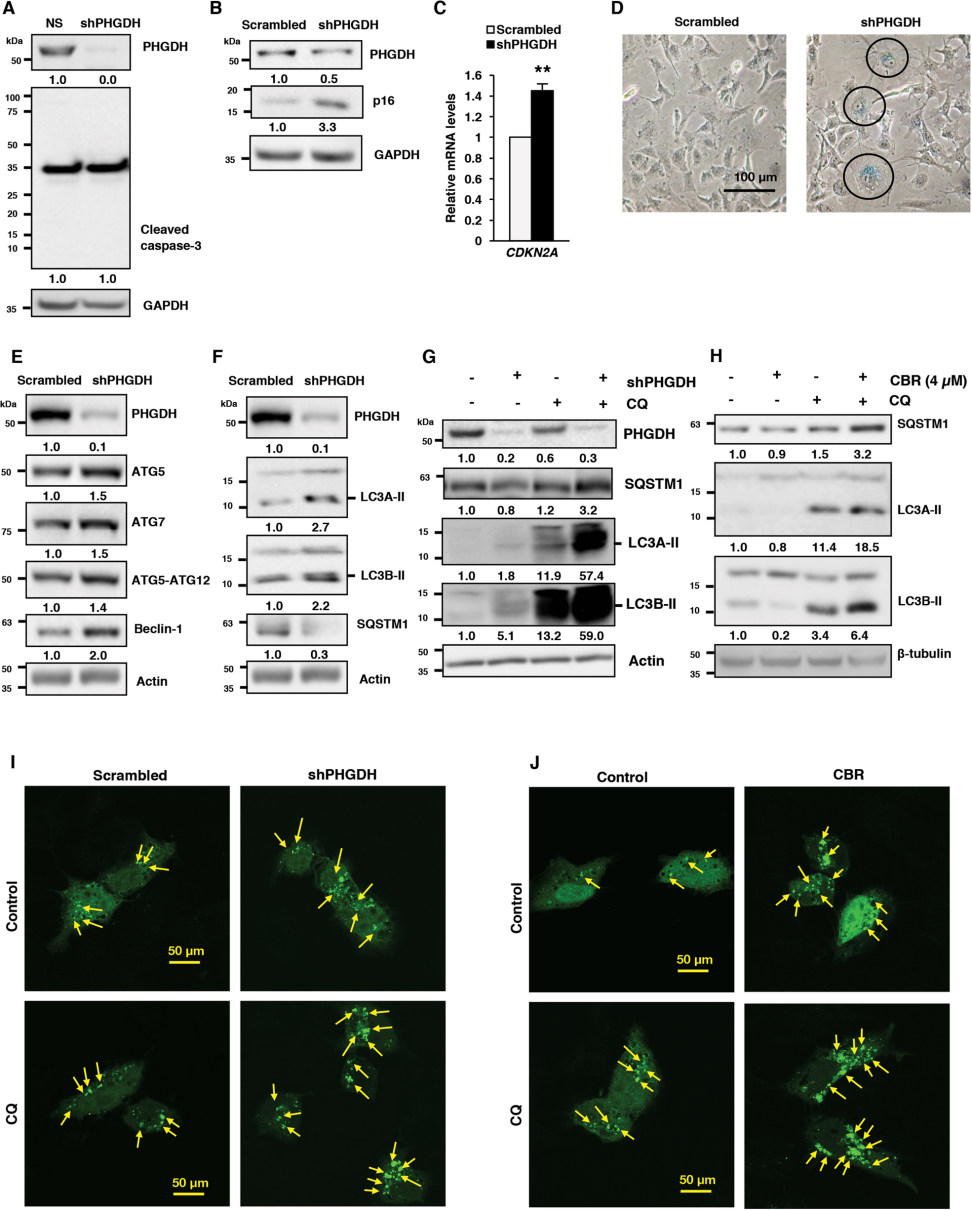
Inhibition of Oct4 and induction of differentiation following PHGDH inhibition is accompanied by autophagy in ECSLCs. **a**–**d** NT2/D1 cells with either scrambled control or PHGDH shRNA were subjected to western blot (WB) analysis for **a** caspase-3, and **b** p16; and **c** qRT-PCR analysis for *CDKN2A;* and **d** β-galactosidase staining. **e** NT2/D1 cells scrambled control or PHGDH shRNA were subjected to WB analysis for ATG5, ATG7, ATG12, Beclin-1; **f** LC3A-II, LC3B-II, and SQSTM1; **g** treated with chloroquine (CQ) and subjected to WB analysis for SQSTM1, LC3A-II, and LC3B-II. **h** NT2/D1 cells were treated with PHGDH inhibitor CBR-5884 (4 µM) alone and in combination with chloroquine (CQ), and subjected to WB analysis for SQSTM1, LC3A-II, and LC3B-II. **i** NT2/D1 cells, with scrambled control or PHGDH shRNA, were treated with LC3-GFP overexpressing plasmid and CQ, alone or in combination, and analyzed by confocal microscopy. **j** NT2/D1 cells, control or LC3-GFP overexpressing, were treated with PHGDH inhibitor CBR-5884 (4 µM) and CQ, alone or in combination, and then analyzed by confocal microscopy. *p ≤ 0.05; **p ≤ 0.01; ***p ≤ 0.001

Next, we dissected the mechanisms governing PHGDH KD-induced effects on cell growth, stemness and differentiation in ECSLCs. Interestingly, PHGDH KD-induced decrease in growth and stemness of ECSLCs did not correspond to an upregulation of apoptotic cell death, as we were unable to detect the presence of cleaved caspase-3 (Fig. 6a). Instead, PHGDH KD in ECSLCs increased the protein (Fig. 6b) and mRNA (Fig. 6c) levels of the tumor suppressor p16^Ink4A^/*CDKN2A*, which inhibits cell cycle progression and induces senescence^36^. Concurring with our observations that PHGDH-depletion upregulated p16^Ink4A^/*CDKN2A*, we observed that PHGDH KD promoted senescence in ECSLCs as demonstrated by an increase in β-galactosidase activity (Fig. 6d)^37^.

Because of the central role of PHGDH in regulating serine metabolism, it is possible that the growth inhibition effects of PHGDH deficiency are related to metabolic stress. In line with this theory, Samanta et al.^28^ have also shown that hypoxia-induced upregulaton of PHGDH expression increases serine levels, whereas PHGDH deficiency reduces serine levels and increases mitochondrial redox stress. Such perturbations in energy metabolism can stimulate fluctuations in autophagy, a catabolic degradation process that is critical for maintaining stemness^21–23^. Our previous work has demonstrated that Oct4 KD-induced differentiation and/or senescence in ECSLCs^22^ is linked with the deregulation of autophagy. Therefore, we hypothesized that PHGDH may modulate autophagy in ECSLCs. First, we found that PHGDH KD in NT2/D1 cells increased the protein (Fig. 6e) and mRNA (Fig. S3A) expression of the molecules involved in autophagy initiation ATG5/*ATG5*, ATG7/*ATG7*, ATG12/*ATG12* and Beclin-1/*BECN1*. Moreover, we observed higher levels of the autophagosome markers LC3A-II and LC3B-II, and decreased levels of the autophagy cargo-targeting protein p62/*SQSTM1* (Fig. 6f) following PHGDH KD in NT2/D1 cells. Using a characteristic flux analysis that monitors the dynamic changes of the autophagic degradation process^27^, we found that treatment with the late-stage autophagy inhibitor chloroquine (CQ) further enhanced the accumulation of LC3A-II, LC3B-II and p62/*SQSTM1* in PHGDH KD NT2/D1 ECSLCs compared to the scrambled control, suggesting PHGDH KD in NT2/D1 ECSLCs enhances autophagic activity (Fig. 6g). To determine whether pharmacological inhibition of PHGDH displayed similar effects on autophagy in ECSLCs, we analyzed autophagy flux following treatment with low (4 µM) and high (10 µM) doses of CBR-5884. Interestingly, we found that low-dose (4 µM) treatment with PHGDH inhibitor mimicked the effect of PHGDH KD and increased autophagy flux in NT2/D1 ECSLCs (Fig. 6h); however, we observed no further upregulation of autophagy flux in cells treated with high-dose (10 µM) of CBR-5884 (Fig. S3B). Upon further analysis, we attributed this lack of additional autophagy upregulation to an increase in apoptosis in NT2/D1 ECSLCs treated with high dose (10 µM) [Fig. S3C (i)], which was absent with low dose (4 µM) [Fig. S3C (ii)], of CBR-5884 as evidenced by increased levels of cleaved caspase-3. Finally, using the LC3-GFP puncta formation assay, visualizing LC3 within autophagic structures^27^, we observed an increase in the number of LC3-puncta following PHGDH KD (Fig. 6i) or low dose (4 µM) of CBR-5884 treatment (Fig. 6j) that was further enhanced by treatment with CQ in ECSLCs. Supplementary Figure S3D presents quantification of number of LC3-GFP puncta formed in NT2/D1 cells with PHGDH KD [Fig. S3D (i)] or treated wth low dose (4 µM) of CBR-5884 [Fig. S3D (ii)]. Altogether these findings, showing that PHGDH deficiency promotes autophagy (not apoptosis), sheds more light on PHGDH-related growth mechanisms in cancer stem-like cells.

### PHGDH inhibition-induced autophagy is p-mTOR-independent, Beclin-1-dependent

**Fig. 7:**
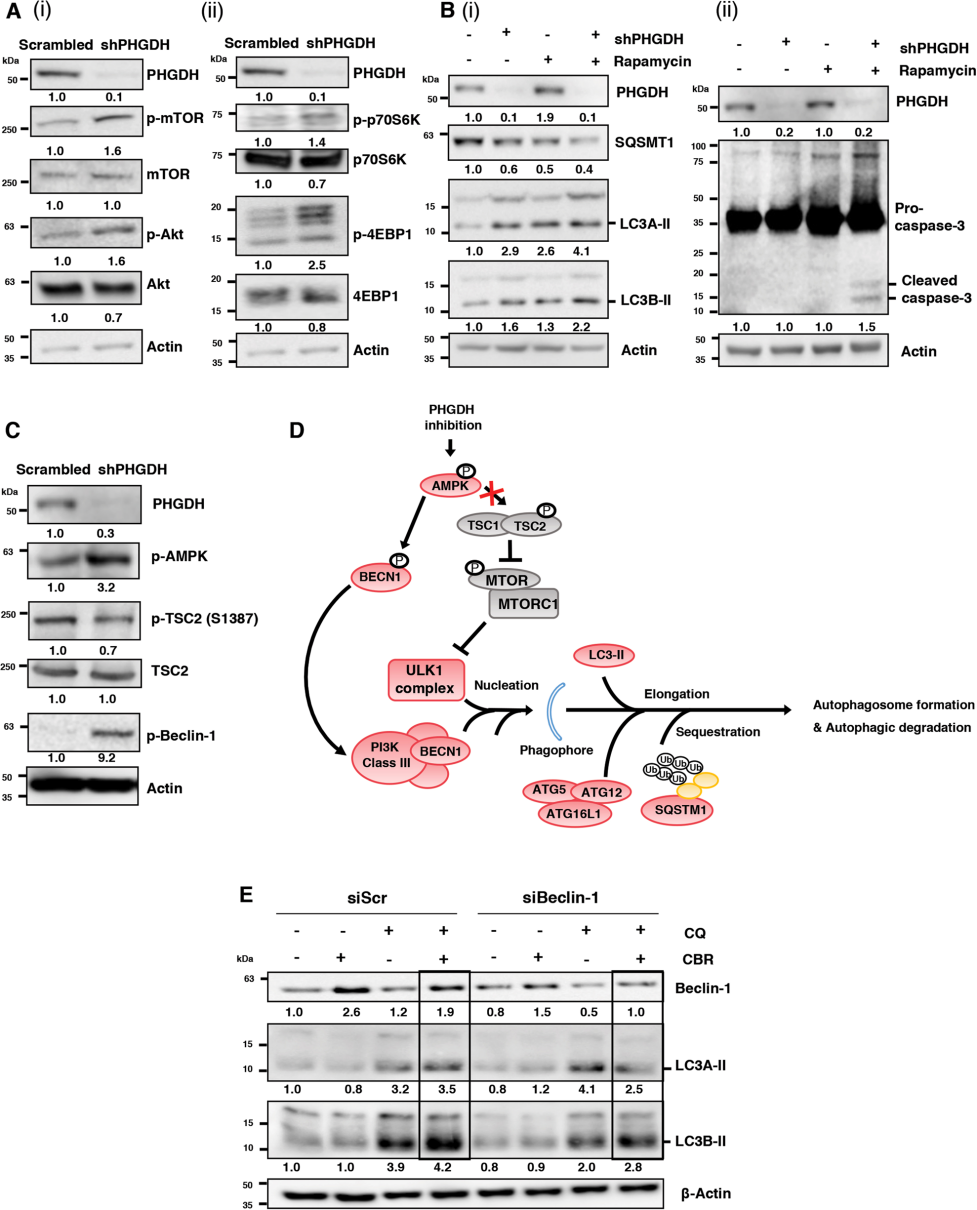
PHGDH inhibition promotes Beclin-1-dependent canonical autophagy mediated by p-AMPK in a p-mTOR-independent manner. **a** NT2/D1 cells, with scrambled control or PHGDH shRNA, were subjected to western blot (WB) analysis for (i) p-mTOR, mTOR, p-Akt and Akt, (ii) p-p70S6K, p70S6K, p-4EBP1, 4EBP1. **b** NT2/D1 cells, with scrambled control or PHGDH shRNA, were treated with rapamycin and subjected to WB analysis for (i) SQSTM1, LC3A-II, LC3B-II, and (ii) caspase-3. **c** NT2/D1 cells, with scrambled control or PHGDH shRNA, were subjected to WB analysis for p-AMPK, p-TSC2, TSC2, p-Beclin-1. **d** Schematic diagram depicting the activation of autophagy through AMPK-Beclin-1 activation due to energy depletion. **e** NT2/D1 cells, with scrambled control or siBeclin-1 siRNA, were treated with PHGDH inhibitor CBR-5884 and chloroquine (CQ), alone or in combination, and the levels of LC3A-II and LC3B-II were analyzed by WB

To understand the mechanism by which PHGDH modulates autophagy in ECSLCs, we probed the mTOR signaling pathway, as phosphorylated mTOR (p-mTOR) is a major negative regulator of autophagy following various stress-inducing stimuli, such as energy and nutrient deprivation^38^. Surprisingly, we found that PHGDH KD increased the levels of p-mTOR [Fig. 7a (i)] and its downstream targets p-p70S6K and p-4EBP1 [Fig. 7a (ii)]^39^. Furthermore, we found that the levels of p-AKT [Fig. 7a (i)], an upstream activator of p-mTOR, were also increased following PHGDH KD^40^. We hypothesized that p-mTOR levels may be upregulated as a feedback mechanism to counter PHGDH KD-induced autophagy in ECSLCs. Therefore, we treated PHDGH KD cells with a p-mTOR inhibitor, Rapamycin, and analyzed the levels of autophagy. We found that combination of PHGDH KD and Rapamycin further enhanced the levels of LC3A-II and LC3B-II and decreased the expression of p62/*SQSTM1* [Fig. 7b (i)], consistent with a cumulative increase in autophagy. Moreover, we found that combination of PHGDH KD and Rapamycin treatment increased the levels of cleaved caspase-3 [Fig. 7b (ii)], indicating an increase in apoptosis. These results were consistent with our hypothesis that upregulation of p-mTOR acts as a rescue mechanism to counter PHGDH KD-mediated effects in ECSLCs.

Given that PHGDH KD does not promote autophagy through inhibition of p-mTOR, we analyzed the expression of other upstream regulators of autophagy. p-AMPK is a positive regulator of autophagy that is increased during energy deprivation and can upregulate autophagy using p-mTOR-dependent or -independent mechanisms^38,41^. p-AMPK can phosphorylate TSC2 at S1387 to activate the tuberous sclerosis complex which inhibits p-mTOR^40,42^. However, p-AMPK can also directly phosphorylate and activate the autophagy-related protein Beclin-1^43^. Phosphorylation of Beclin-1 by p-AMPK promotes its association with the ULK1 complex and promotes autophagy initiation and phagophore nucleation^43^. As shown in Fig. 7c, the levels of p-AMPK increased following PHGDH KD, but this upregulation of p-AMPK was not accompanied by an increase in the levels of p-TSC2. This is consistent with our previous observations that PHGDH KD does not inhibit p-mTOR to promote autophagy. Instead, we found that PHDGH KD increased the levels of p-Beclin-1 (Fig. 7c), indicating that PHGDH KD-mediated upregulation of autophagy is mediated by p-AMPK through phosphorylation of Beclin-1 in a p-mTOR-independent manner.

Because we observed that PHGDH depletion upregulates p-AMPK and p-Beclin-1 expression ECSLCs, we next determined whether this upregulation of p-Beclin-1 was responsible for PHGDH KD-mediated autophagy. As illustrated in Fig. 7d, Beclin-1 activation is required for the induction of canonical autophagy, which involves de novo phagophore nucleation and lipidation to form double membrane autophagic vacuoles^44^. Other types of autophagy include non-canonical autophagy, where pre-existing double membrane structures can be used to form autophagic vacuoles in a manner that is independent of Beclin-1^44^. To conclusively demonstrate whether autophagy induced by PHGDH inhibition relies on the Beclin-1-dependent canonical autophagy pathway, we treated NT2/D1 ECSLCs with the PHGDH inhibitor CBR-5884 in combination with CQ and Beclin-1-specific siRNA. We found that inhibition of Beclin-1 partially rescues the upregulation of autophagic flux induced by CBR-5884 (comparison between lanes 4 and 8 in Fig. 7e), suggesting that PHGDH inhibition promotes autophagy that is, in part, dependent on Beclin-1-mediated induction of canonical autophagy. Altogether, these findings demonstrate that PHGDH related effects on autophagy are independent of p-mTOR.

## Discussion

Previous studies have demonstrated that several types of cancerous cells harbor high levels of PHGDH compared to normal cells. In particular, a recent study by Zhang et al.^7^ has shown that PHGDH is amplified in some lung carcinoma cells and that this overexpression of PHGDH is linked with rapid proliferation. Furthermore, this study also demonstrated that depletion of PHGDH has a more pronounced effect on growth inhibition in cancer cells with amplified PHGDH expression^7^. Similar observations have been confirmed in other models of cancer. Possemato et al.^5^ have previously shown that PHGDH is amplified in ER-breast cancer, and that these cells are more sensitive to growth suppression by PHGDH inhibition than cancer cells without PHGDH overexpression^5^. Our findings demonstrating that PHGDH expression is higher in CSLCs (CD133^High^ BTICs, HMLER^shECad^ BCSLCs, and NT2/D1 ECSLCs) compared to various other non-stem-like cancer cells further corroborate these previous reports linking PHGDH with cancer aggressiveness, further supporting the clinical implications for PHGDH in cancer therapy.

PHGDH has recently emerged as an important regulator of stemness in cancer, as demonstrated by a recent publication by Samanta et al.^28^ which showed that PHGDH is required for maintaining BCSCs induced by hypoxia. In this report, we demonstrate a novel link between PHGDH and self-renewal, stemness-maintaining transcription factors (Oct4, Nanog, Sox-2, KLF4, and Lin28b), differentiation, and autophagy in CSLCs. In addition to regulating the transcription of several differentiation markers, we also show that PHDGH differentially modulates the stability of the major factors that play pivotal role in differentiation and stemness through post-translational modifications. While PHGDH KD increases the specific ubiquitination and subsequent proteasomal degradation of stemness-maintaining factor Oct4, in contrast, it promotes the post-translational stabilization of β3-tubulin by decreasing its specific ubiquitination. Collectively, these data report contrasting effects of PHGDH on the characteristic molecular targets of differentiation and stemness features in CSLCs.

CSLCs are a small population of poorly differentiated cells that reside within heterogeneous tumor masses^13^. In line with their capacity of self-renewal and multilineage differentiation, these CSLCs have been shown to fuel the tumor formation^16,45,46^, and often resort to aberrant metabolic pathways to sustain their continuous demand for energy and macromolecules^47^. Hence, it is reasonable to speculate that the introduction of defects causing metabolic inefficacies in CSLCs would be harmful for their maintenance and growth. In support of this hypothesis, the recent reports have shown that the stemness-regulating transcription factors, such as Oct4 and Nanog, are linked with metabolic reprogramming to support the growth of normal and cancer stem cells^48–50^. Interestingly, strategies promoting differentiation in CSLCs are being acknowledged for their therapeutic potential, as the differentiated cells can be more susceptible to various anti-cancer treatment options as compared to the CSLCs^15,19,20^. Thus, further understanding of the relationship between stemness, differentiation and metabolism is important for the development of novel therapeutic strategies targeting CSLCs. Here, we show that serine biosynthesis enzyme PHGDH differentially regulates the factors involved in the regulation of stemness and differentiation of CSLCs.

Screening of large datasets-based gene expression profiles of patient-derived tumor samples is a useful tool to identify the molecular pathways linked with CSLC biology. In order to explore the link of PHGDH with stemness, we analyzed gene expression datasets from human tumors and discovered a clear correlation between PHGDH and stemness factors Oct4, CD133, MYC, and SOX9. Most importantly, the clinical relevance regarding the correlation between PHGDH and Oct4 is demonstrated by the finding that patients with tumors co-expressing high levels of PHGDH and Oct4 have significantly lower survival prognosis than those with the low co-expression. These findings strongly support our hypothesis that PHGDH supports the stemness features in CSLCs. It should be noted that, when we performed a screening for PHGDH and Oct4 co-expression in a variety of cancerous cells of various origins and BTICs, we found that NT2/D1 ECSLCs expressed the highest levels of PHGDH and were the only cell line to co-express PHGDH and Oct4. Considering the clinically relevant link between PHGDH/Oct4 co-expression and patient survival, NT2/D1 teratocarcinoma cells with stem-like features will stimulate further research into the therapeutic utility of PHGDH–Oct4 axis.

We found that CSLCs rely on PHGDH levels to maintain their growth. PHGDH KD in CSLCs strongly lowers the expression of stemness-regulating factors such as Oct4, Nanog, Sox-2, KLF4, Lin28b, and Bmi-1, and reduces their self-renewal ability as evidenced by the decreased tumorsphere formation capacity. Interestingly, this decreased stemness leads to multilineage differentiation in ECSLCs. Mechanistically, we showed that PHGDH inhibition promotes differentiation by differentially regulating the stability of Oct4 and β3-tubulin proteins. In line with the recent reports demonstrating the connection between loss of stemness, for example through Oct4 KD, and the resultant aberrations within autophagic homeostasis^22^, we found that PHGDH inhibition-related effects on self-renewal and differentiation are also linked with autophagy induction. We found that PHGDH KD-induced autophagy involves p-mTOR-independent and Beclin-1-dependent mechanisms. Through inhibition of p-mTOR via Rapamycin in PHGDH-KD ECSLCs, we were able to further enhance autophagy upregulation and promote apoptosis as evidenced by the activation of cleaved caspase-3. In the context of the emerging appreciation for autophagy in stemness maintenance^21–23^, these findings provide an interesting link between PHGDH, autophagy and stemness, and warrant the need for exploring the anti-cancer therapeutic potential of autophagy-inducing and differentiation-promoting metabolic strategies.

In summary, our study shows that PHGDH supports self-renewal and poorly differentiated form in ECSLCs. Given the emerging interest in the differentiation-based therapies to target CSLCs, our study identifies PHGDH inhibition as a strategy to promote therapeutically desired differentiation in CSLCs.

## Materials and methods

### Cell culture and treatments

NT2/D1 cells were maintained in DMEM, HMLE, and HMLER were maintained in DMEM F12 while HMLER^shECad^ were maintained in serum-free HUMEC Ready Media (Gibco). DMEM media was supplemented with 10% heat-inactivated FBS, 1% penicillin/streptomycin, and 1% non-essential amino acids (ThermoFisher). DMEM F12 was supplemented with 5% heat-inactivated FBS, 20 ng/mL EGF, 10 µg/mL insulin, 0.5 µg/mL hydrocortisone and 1% penicillin/streptomycin. For autophagy flux assays, actively growing cells were treated with 12 μM of chloroquine for 24 h. For siRNA treatment cells were transfected with 50 nM of siPHGDH (ThermoFisher, AM16708; Assay ID: 108071; accession number: NM_006623.3) for 24 h. For siRNA flux assay, cells were treated with 50 nM of siRNA against Beclin-1 (Cell Signaling, 6246; Entrez Gene ID: 8678) using Lipofectamine (Invitrogen) for 24 h before treating with 4 µM of CBR-5884 (ApexBio, A8721) and/or 12 µM of chloroquine (CQ). Lysate was collected after another 24 h.

### Dissociation and culture of primary GBM tissue

Human GBM samples were obtained from consenting patients, as approved by the Hamilton Health Sciences/McMaster Health Sciences Research Ethics Board. Tumor tissues were dissociated in PBS containing 0.2 Wünsch unit/mL Liberase Blendzyme 3, and incubated at 37 °C in a shaker for 15 min. The dissociated tissue was filtered through a 70 µm cell strainer and collected by centrifugation (450 × *g*, 3 min). Red blood cells were lysed using ammonium chloride solution (STEMcell Technologies). The cells were washed with PBS, and resuspended in NeuroCult™ NS-A Proliferation Medium (STEMcell Technologies) supplemented with epidermal growth factor (20 ng/mL), basic fibroblast growth factor (10 ng/mL), 2 μg/mL of Heparin and antibiotic–antimycotic (1× Wisent). The cells were then plated on ultra-low attachment plates (Corning) and propagated as tumorspheres.

### Flow cytometric analysis of GBM BTICs

Tumorspheres were suspended in 1 mL of PBS, dissociated into single cells using Liberase Blendzyme 3, washed with PBS, resuspended in PBS + 2 mM EDTA and filtered through 35 μm filter. Single-cell suspensions were stained with APC-conjugated anti-CD133 or a matched isotype control (Miltenyi) as recommended by the manufacturer and incubated for 15 min at room temperature. Samples were run on a MoFlo XDP Cell Sorter (Beckman Coulter). Dead cells were excluded using the viability dye 7AAD (1%; Beckman Coulter). Compensation was performed using mouse IgG CompBeads (BD Biosciences). Expression of CD133 was defined as positive or negative based on the analysis regions set on the isotype control.

### Tumorsphere assays

Tumorsphere formation was induced using ultralow-adherent six-well plates. Cells were seeded at a density of 60,000 cells per well for NT2/D1 and 40,000 cells per well for HMLER^shECad^ in a serum-free HUMEC Ready Media for stem cells (Gibco). Tumorsphere formation was quantified 4 and 7 days after initial seeding using ImageJ. Spheres with a diameter equal or higher than 50 µm were deemed tumorspheres and average number of tumorspheres per 10^5^ µm of plate surface area was determined. Data are representative of at least three independent experiments and quantified from more than three microscopic fields of view per experiment.

### Lentiviral generation and transduction of NT2/D1 cells

Lentiviral vectors with shRNA sequence targeting PHGDH (GE Dharmacon, clone IDs: TRCN0000041626 and TRCN0000041627; sequences: shPHGDH #1: ATCAGCAGTGACCTTAGTAGC, shPHGDH #2: TTAGCGTTCACCAAGTTCACG), Oct4 (GE Dharmacon, clone ID: TRCN0000004879; sequence: AATTCCTTCCTTAGTGAATGA) or ATG7 (GE Dharmacon, clone ID: TRCN0000007584; sequence: ATGGAGAGCTCCTCAGCAGGC) were purchased from GE Dharmacon. A non-silencing shRNA vector was also used as a control, denoted as scrambled. The envelope plasmid pMD2G and the packaging plasmid psPAX2 were obtained from Addgene. For lentiviral production, 293T cells were co-transfected with the lentiviral expression vector and the packaging DNAs by polyethylenimine (PEI). The supernatant containing lentiviral particles was collected 48 h after transfection. Stable cell lines were established using the lentiviral particles for transduction and selecting cells with puromycin 24 h after transduction.

### Cell viability

Equal numbers of cells from each sample were seeded in six-well plates containing 2 mL of culture medium. After 24 h incubation, cells were treated with chemicals at the indicated concentrations. Adherent cells were dissociated at the indicated times with 0.05% trypsin-EDTA and then counted by trypan blue dye exclusion. The numbers of viable cells are presented as mean ± S.D. of three replicates for each sample.

### Western immunoblotting

Cells were lysed in RIPA buffer (25 mM Tris pH 7.6, 150 mM NaCl, 1% NP-40, 1% sodium deoxycholate, 1% SDS) containing protease inhibitors (ThermoFisher). Protein concentrations were measured using the Micro BCA protein assay kit (ThermoFisher). Equal amount of protein was boiled in Laemmli sample buffer (BioRad) containing 5% β-mercaptoethanol for 5 min and then resolved by SDS-PAGE. Protein was transferred onto nitrocellulose membranes (BioRad). Specific primary antibodies against the following proteins were used for immunoblotting: PHGDH, Oct4 (Santa Cruz Biotechnology, sc-5279), Nanog (Cell Signaling, 4903), Sox-2 (Cell Signaling, 2748), E-cadherin (Cell Signaling, 3195), N-cadherin (Cell Signaling, 13116), Bmi-1 (Cell Signaling, 6964), β3-tubulin (Santa Cruz, sc-80005), β-actin (Santa Cruz, sc-47778), caspase-3, SQSTM1 (Cell Signaling, 5114), LC3A (Cell Signaling, 4599), LC3B (Cell Signaling, 3868), p-mTOR (Cell Signaling, 2971), mTOR (Cell Signaling, 2983), p-p70S6K (Cell Signaling, 9204), p70S6K (Cell Signaling, 2708), p-4EBP1 (Cell Signaling, 2855), 4EBP1 (Cell Signaling, 9644), p-Akt (Cell Signaling, 4060), Akt (Cell Signaling, 2920), p-Beclin-1 (Cell Signaling, 14717), Beclin-1 (Cell Signaling, 3495), p-AMPK (Cell Signaling, 2537), p-TSC2 (Cell Signaling, 23402), TSC2 (Cell Signaling, 4308), p16 (Santa Cruz, sc-390485), ATG5 (Cell Signaling, 8540), ATG7 (Cell Signaling, 8558), Ubiquitin (Santa Cruz, sc-8017), GAPDH (Santa Cruz, sc-365062), and β-tubulin (Cell Signaling, 2146). Secondary antibodies: HRP-conjugated anti-mouse IgG (Jackson ImmunoResearch) and HRP-conjugated anti-rabbit IgG (Jackson ImmunoResearch). Detection was by chemiluminescence (ECL, BioRad) using ChemiDoc Touch Imaging System (BioRad). Quantification was by densitometry using ImageJ software (National Institutes of Health).

### Quantitative real-time PCR analysis

RNA was extracted from cultured cells using Trizol and cDNA was synthesized using enzyme Superscript II (ThermoFisher). Each sample of cDNA was quantitated and diluted to a similar concentration of 10 ng/mL. The BioRad CFX96 PCR machine was used for the quantitative real-time PCR (qRT-PCR), using SYBR Green Supermix (BioRad). All primers were purchased from Invitrogen. GAPDH was used for normalization of the genes of interest. The results were analyzed using 2^−ΔΔCT^ method and expressed as fold change to respective non-treated or scrambled controls.

### Senescence detection

Senescence was detected by using the senescence β-Galactosidase staining kit (Cell Signaling) following the manufacturer’s protocol. The growth media was removed from the cells and the plate was washed one time with 1× PBS (ThermoFisher). Cells were fixed with the 1× Fixative solution and incubated for 15 min at room temperature. After the incubation, the plate was rinsed two times with 1× PBS and 1 mL of the β-Galactosidase Staining Solution was added to each plate. The plate was sealed with parafilm and incubated at 37 °C in a dry incubator. After 24 h, the images were captured using a light microscope.

### Bioinformatics analysis

Normalized data files for GSE25066 were downloaded from GEO database. Correlations were performed between PHGDH and OCT4, MYC, SOX9, CD133, and statistics (correlation index, *R* and *p*-value) were calculated using Pearson correlation method. Kaplan-Meier survival plots were generated with overall survival data and statistics (HR and *p-*value) were generated using log rank test. Graphs were generated using Graphpad Prism software.

### Microscopy and imaging

For puncta formation, cells were plated on coverslips and transfected with 4 µg/ml of EGFP-LC3 plasmid (Addgene, 11546) 24 h prior to CBR and/or CQ treatment. After 24 h, cells were fixed using 4% paraformaldehyde and mounted using mounting medium (Dako, S3023). For β3-tubulin immunofluorescence, cells were plated on coverslips and after 24 h, cells were fixed using 4% paraformaldehyde for 15 min, permeabilized with 0.1% Triton X-100 in PBS and blocked in 1% bovine serum albumin (BSA) for 30 min at room temperature. After rinsing, the cells were incubated with a mouse anti-β3-tubulin antibody (Santa Cruz, sc-80005), diluted 1:250 in 2% BSA. After overnight incubation at 4 °C, the cells were washed with PBS and incubated in goat anti-mouse conjugated to Alexa Fluor 488 (Invitrogen, A-11001), diluted 1:2000 in 2% BSA. After rinsing, cells were stained for an additional 30 min with TO-PRO3 (Life Technologies, T3605), diluted at 1:1000 in PBS. Cells were then washed and mounted using mounting medium (Dako, S3023). Imaging was performed using Zeiss LSM 510.

### Statistical analysis

All values are expressed as mean ± S.E.M. of three independent experiments. Statistical evaluation was performed with using two-tailed, Student’s *t*-test with 95% confidence interval. *p* < 0.05 was considered as significant.

## Electronic supplementary material

Suppl 1

Suppl 2

Suppl 3
