# Supplementary figures and images for: Phosphoglycerate dehydrogenase inhibition induces p-mTOR-independent autophagy and promotes multilineage differentiation in embryonal carcinoma stem-like cells
Source: Cell Death Dis. 2018 Sep 24;9(10):990. doi: 10.1038/s41419-018-0997-8 (PMC6155240; doi:10.1038/s41419-018-0997-8)

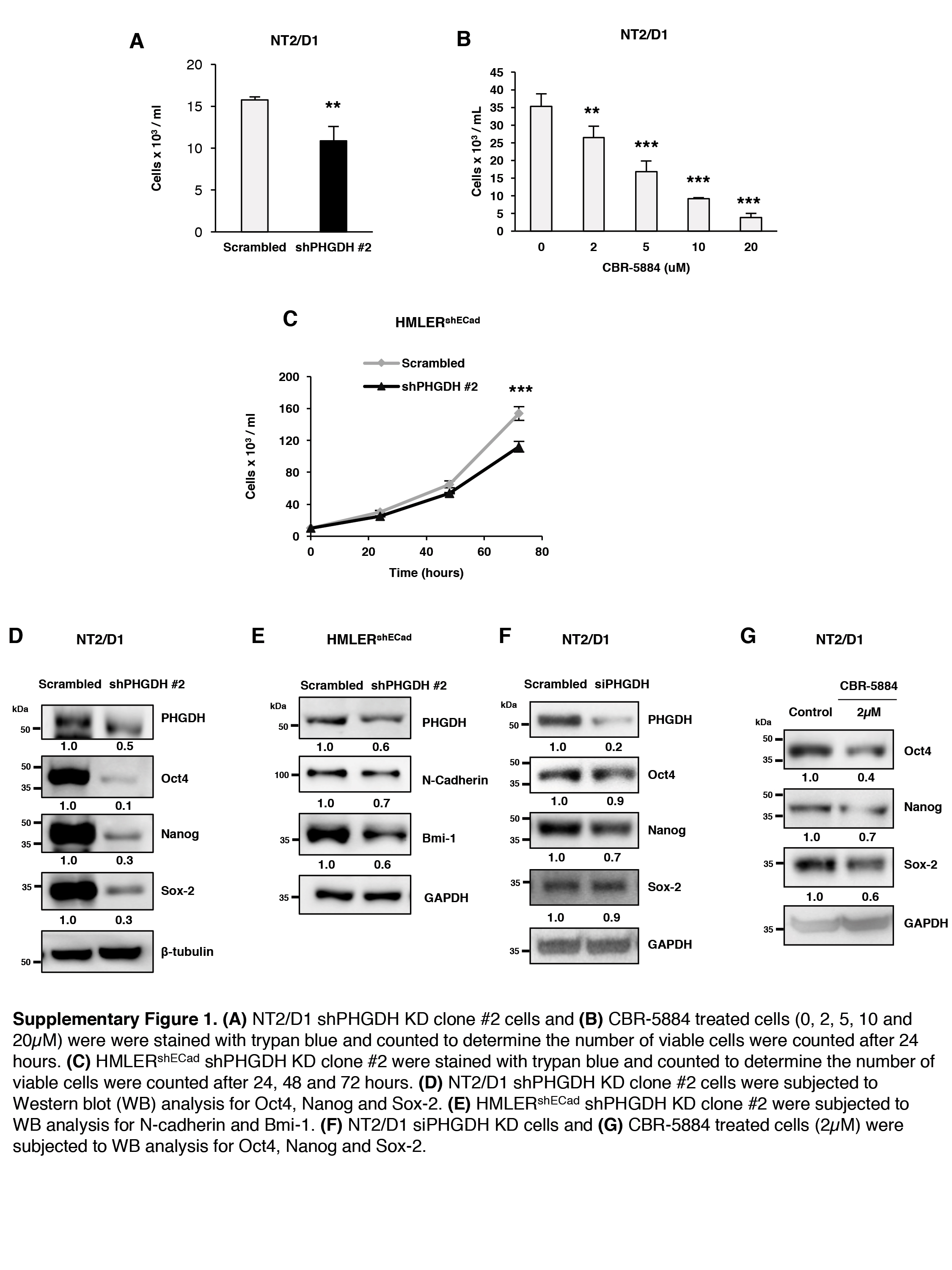

Supplement: Supplementary file 1 — Suppl 1 [file 41419_2018_997_MOESM1_ESM.tif]

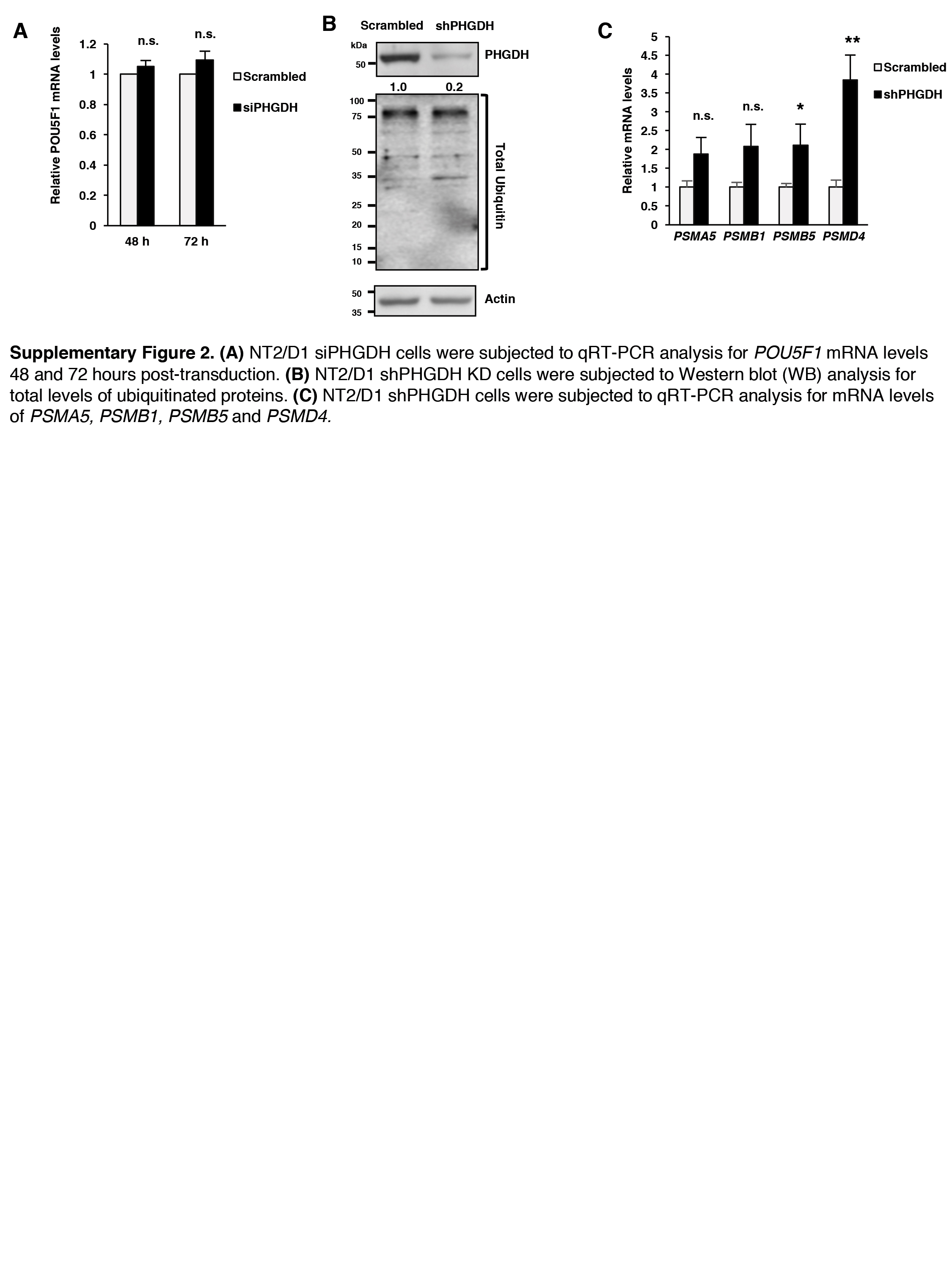

Supplement: Supplementary file 2 — Suppl 2 [file 41419_2018_997_MOESM2_ESM.tif]

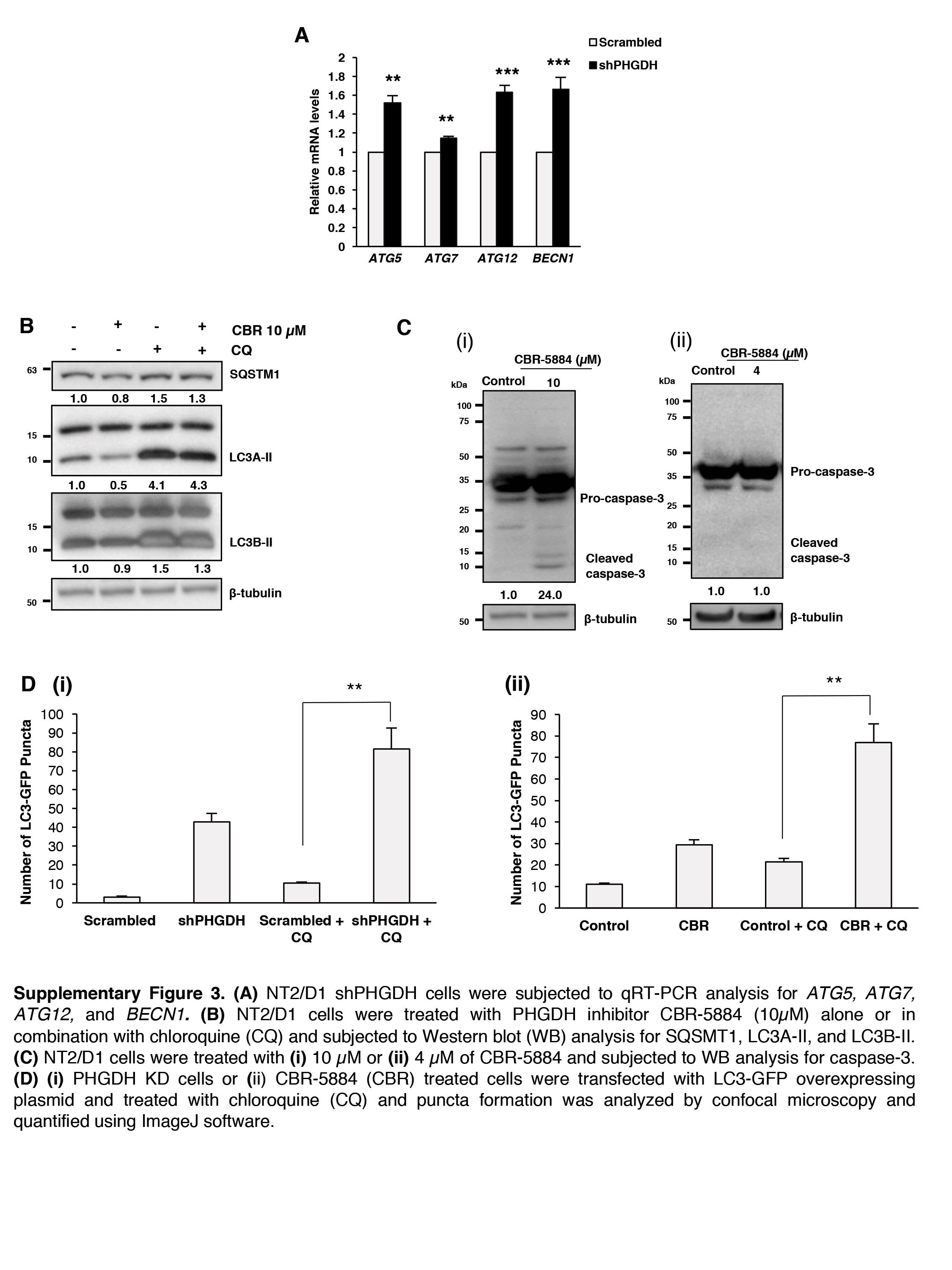

Supplement: Supplementary file 3 — Suppl 3 [file 41419_2018_997_MOESM3_ESM.tif]
